# Development of controlled nanosphere lithography technology

**DOI:** 10.1038/s41598-023-29077-y

**Published:** 2023-02-27

**Authors:** Artem A. Osipov, Alina E. Gagaeva, Anastasiya B. Speshilova, Ekaterina V. Endiiarova, Polina G. Bespalova, Armenak A. Osipov, Ilya A. Belyanov, Kirill S. Tyurikov, Irina A. Tyurikova, Sergey E. Alexandrov

**Affiliations:** 1 Peter the Great St. Petersburg Polytechnic University, St. Petersburg, 195251 Russian Federation; 2 Institute of Mineralogy of Southern-Urals Federal Research Center of Mineralogy and Geoecology of Ural Branch of RAS, Miass, Chelyabinsk Region 456317 Russian Federation

**Keywords:** Self-assembly, Surface patterning, Nanoparticles, Nanowires

## Abstract

This work is devoted to the development of nanosphere lithography (NSL) technology, which is a low-cost and efficient method to form nanostructures for nanoelectronics, as well as optoelectronic, plasmonic and photovoltaic applications. Creating a nanosphere mask by spin-coating is a promising, but not sufficiently studied method, requiring a large experimental base for different sizes of nanospheres. So, in this work, we investigated the influence of the technological parameters of NSL by spin-coating on the substrate coverage area by a monolayer of nanospheres with a diameter of 300 nm. It was found that the coverage area increases with decreasing spin speed and time, isopropyl and propylene glycol content, and with increasing the content of nanospheres in solution. Moreover, the process of controllably reducing the size of nanospheres in inductively coupled oxygen plasma was studied in detail. It was determined that increasing the oxygen flow rate from 9 to 15 sccm does not change the polystyrene etching rate, whereas changing the high-frequency power from 250 to 500 W increases the etching rate and allows us to control the decreasing diameter with high accuracy. Based on the experimental data, the optimal technological parameters of NSL were selected and the nanosphere mask on Si substrate was created with coverage area of 97.8% and process reproducibility of 98.6%. Subsequently reducing the nanosphere diameter lets us obtain nanoneedles of various sizes, which can be used in field emission cathodes. In this work, the reduction of nanosphere size, silicon etching, and removal of polystyrene residues occurred in unified continuous process of plasma etching without sample unloading to atmosphere.

## Introduction

The creation of ordered arrays of silicon nanostructures is of great interest to researchers due to their unique properties and potential applications in various components of electronic^1,2^, plasmonic^3,4^, photonic^5^, photovoltaic^6,7^ devices, as well as surface-enhanced Raman spectroscopy (SERS)^8^. For example, the authors of^9^ created a prototype of vertically integrated nanowire field effect transistors (SiNW-FETs) based on vertically oriented silicon structures. In addition, SiNW-FETs are promising for biosensors due to their ultrasensitivity, selectivity, and label-free and real-time detection capabilities^10^. It is also known that the solar cells made on the basis of silicon vertically oriented nanostructures are promising in the solar energy industry. This is because of such advantages as increased efficiency of light trapping due to its multiple scattering inside the structure, with much lower mass and thickness of the cell compared to planar cells^11^. Another promising application of silicon vertically oriented nanostructures, namely nanoneedles, is their use as field emission (cold) cathodes in electrovacuum devices. In contrast to thermionic cathodes, in this case electrons do not need to be pre-excited for their emission under the action of electric field^12,13^.

However, one of the main problems of technology development in this direction is the lack of simple methods for forming a pattern on the substrate surface to obtain the required topology. Typically, traditional lithographic techniques such as extreme ultraviolet (EUV) lithography and electron beam lithography (EBL) combined with a dry plasma etching process are used to create nanostructures with controllable size and shape. For example, in^14^ the authors demonstrate their refractive index sensor based on an array of resonant silicon nanodisks with diameter of 330 nm, which were fabricated using EBL and reactive ion etching. In another paper^15^, the authors present a process for fabricating an array of metallic nanowires smaller than 100 nm in diameter using EUV and plasma etching in oxygen. Despite the fact that the use of short-wavelength radiation and an alternative method of exposure made it possible to reduce the size of the obtained structures, these methods are characterized by significant financial and time expenditures, as well as technical complexity in their realization^16^. In this regard, the search and study of simpler, cheaper and more productive approaches to the formation of nanostructures with certain parameters has become of primary relevance and practical significance.

The development of nanosphere lithography based on the phenomenon of self-organization has established itself as an alternative method for creating ordered nanostructures with such advantages as low cost, high productivity and lack of complex equipment^17^. The most common material for nanospheres, playing the role of a mask during subsequent etching, is polystyrene (PS), which is a monodisperse spherical particles in a wide range of sizes (from 20 nm to 10 μm). Self-assembly at the air/water interface (Langmuir–Blodgett (LB) method)^18^ and spin-coating method^19^ are considered to be the most promising techniques for the formation of nanosphere coatings. However, the authors of works^19–21^ note that low transfer rate of nanospheres to the substrate and difficulty in obtaining uniform monolayer coating on a large area prevent wide application of LB method. Coating the substrate using spin-coating method involves deposition of a colloidal suspension to a hydrophilic substrate followed by an accelerated solvent evaporation process during spinning in a centrifuge. This method is currently considered the most efficient and flexibly controlled way for creating monolayers of self-assembled particles, which can be implemented in mass production. In addition, in the research^22^ authors studied nanospheres of size 500 and 1000 nm to compare spin-coating and LB methods, and they found that in the case of using nanospheres of small diameter the LB method does not let to obtain a close-packed monolayer on the whole substrate surface (the maximum coverage area was 54%).

However, spin-coating method is not simple, because it requires optimization of a number of parameters that have an interdependent influence on the solvent evaporation process. In this regard, researchers have to find optimal technological parameters empirically, and usually the optimal mode varies depending on the size of nanospheres. To date, the application of spin-coating method for the formation of nanosphere coatings can be found in many papers. However, most of them either do not give any detailed information on the selection of optimal technological parameters, or study the influence of only some parameters on the quality of the resulting coating. For example, the authors of^21^ show how spin speed and concentration of the dispensed colloidal solution affect the process of creating an ordered array of nanospheres with different diameters. But in this study the focus was placed exclusively on achieving perfect orderliness on small-area samples (15 × 15 mm), and the effect of technological parameters on the total coverage area of the entire substrate was not investigated. In another study^20^, Chen et al. found a correlation between the area covered by a mono- and bilayer of nanospheres and parameters of the process such as spin speed and acceleration for four different sizes of nanospheres. However, in this work, the authors did not study the parameters related directly to colloidal solution preparation, which have a significant influence on the solvent evaporation process and hence on the quality of nanosphere self-assembly. In addition, it is worth noting that in some works^21,23–25^, mixtures of surfactants were additionally added to the solution with nanospheres to improve the wettability of the solution and slow down the evaporation rate of the suspension. But this fact can not only lead to additional contamination of the substrate with surfactants, but also significantly increase the cost of the nanosphere lithography process. It is important that in most works aimed at studying the spin-coating process, microphotographs demonstrating obtained nanosphere coatings cover the substrate area of no more than 100 × 100 µm^2^^19,20,22,23,26^. Such magnification does not allow to objectively estimate the filling and ordering of nanospheres in larger areas, whereas high uniformity and monolayer coverage over the entire substrate area is a fundamental requirement for the nanosphere lithography process if it is used to create etching masks. Thus, a detailed investigation of the influence of technological parameters on the process of creating a monolayer uniform nanosphere coating is a relevant task, and it can facilitate the implementation of the nanosphere lithography by spin-coating method in both scientific and industrial fields.

After obtaining a close-packed array of nanospheres on the substrate, it can be used immediately as a mask for the etching process or modified in a certain way to obtain the desired pattern. Plasma-chemical etching (PCE) in oxygen is widely used to reduce the size of nanospheres without changing their position on the substrate^27–30^. The size of nanospheres can be controlled by adjusting the duration and conditions of etching, and the distance between them depends on the choice of the initial size of the spheres. At the same time, the creation of silicon nanostructures through a mask of nanospheres in a single etching cycle is of practical interest. It implies reduction of nanosphere size, etching of silicon and removal of nanosphere residues in a single process chamber without additional stages of sample loading and unloading to atmosphere. In this regard, this work is devoted to the development of the technology of nanosphere lithography (nanospheres with a diameter of 300 nm) using spin-coating method, the study of the influence of technological parameters on the process of reducing the size of spheres in plasma and approbation of the developed technology to create an array of silicon nanoneedles of a certain size in a single cycle for their potential use in the manufacture of field emission cathodes.

## Methods

Monodisperse polystyrene nanospheres with a diameter of 300 nm in the form of an aqueous solution with a dry substance concentration of 10 wt % were purchased from the Institute of High-Molecular Compounds of the Russian Academy of Sciences and were used for the experiments. Monocrystalline silicon wafers (100) with a diameter of 76 mm and a thickness of 380 μm were purchased from OOO "Kremni" and were used as samples for depositing nanospheres. Before each experiment, the substrates were cleaned in acetone for 10 min at 80℃. Due to the fact that nanosphere lithography requires a hydrophilic surface, before nanosphere deposition, additional treatment of the sample surface in a piranha solution (1:3 H_2_O_2_:H_2_SO_4_) followed by washing in distilled water and drying in an air stream was applied.

Spin-coating process was conducted on a JOANLAB MC-12 Pro high-speed centrifuge. The process of nanosphere lithography included the following steps:

1. Taking the required amount of aqueous solution of polystyrene nanospheres using a micropipette (JOANLAB) and placing it in a test tube for centrifugation.
2. Centrifugation at 12,000 rpm for 8 min to separate the solution into fractions followed by removal of excess water from the test tube.
3. Adding the necessary amount of isopropyl alcohol and propylene glycol to the test tube to increase the viscosity of the solution.
4. Ultrasonic treatment of the resulting solution for 15 min at 50 ℃ for mixing the solution and uniform distributing the nanospheres in the suspension to prevent their sticking together.
5. Transferring the prepared solution to the hydrophilic substrate using the micropipette and spin-coating at a certain speed and time. The acceleration of rotation was not regulated due to the lack of this function in the centrifuge.
6. Drying the wafer on an oven at 60 ℃ for 5 min to finally evaporate the solvent and provide better contact between the nanospheres and the Si substrate.

**Figure 1:**
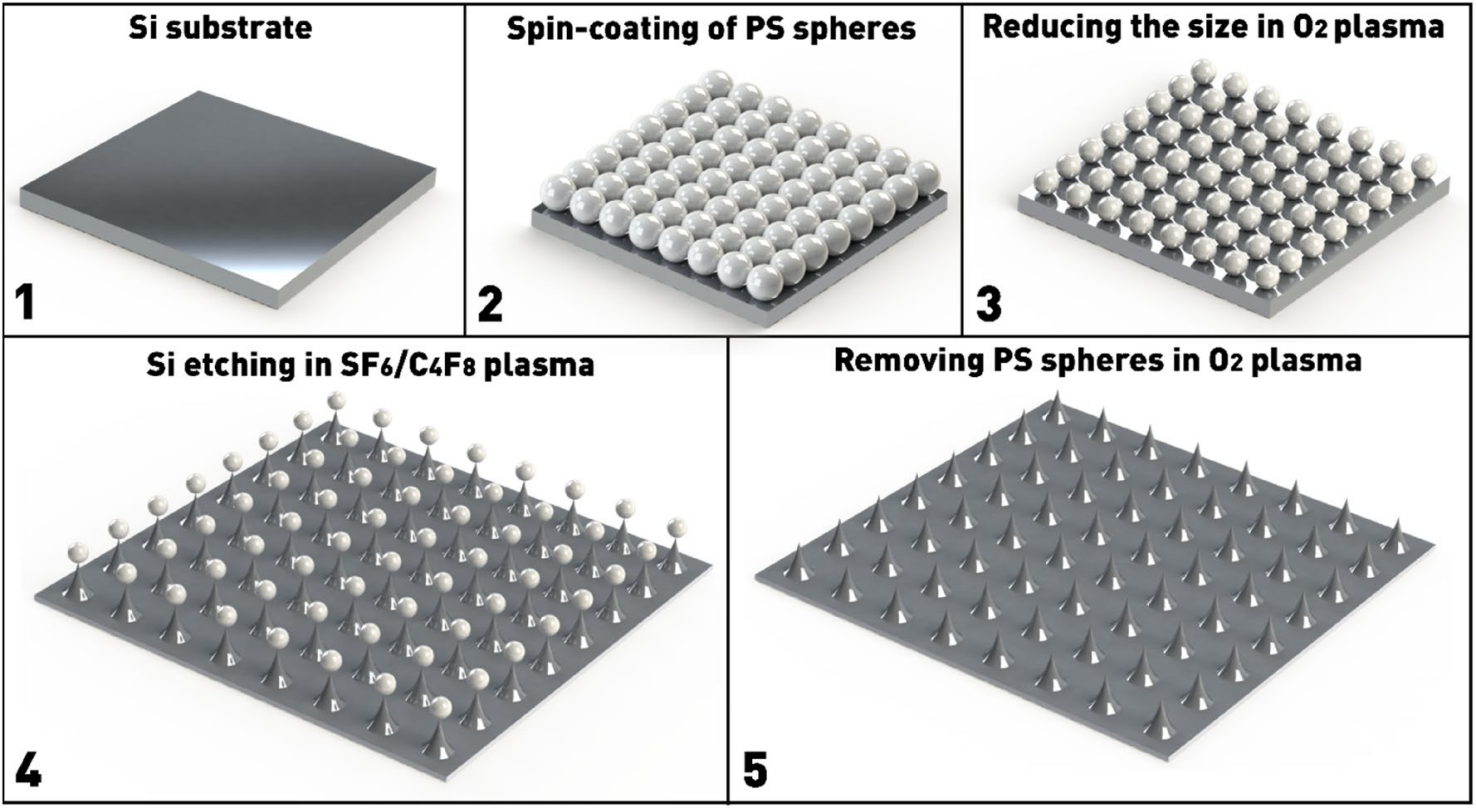
Schematic image of the basic steps for creating ordered silicon nanostructures through a mask of polystyrene nanospheres.

Reducing the size of nanospheres and subsequent etching of silicon through the resulting mask was performed on a custom-built plasma-chemical etching system with a source of high-density inductively coupled plasma (ICP), described in details previously^31^. Oxygen (O_2_ class 6.0, 99.9999%, GOST TU 2114-001-05798345-2007) was used for isotropic etching of polystyrene nanosphere in ICP, first to reduce the diameter of nanospheres in the range from 300 to 15 nm, and at the end of the process to remove residual nanospheres. The Si substrate with a monolayer of polystyrene nanospheres as a mask was etched in ICP using a gas mixture of SF_6_ (99.998%, GOST TU 6-02-1249-83) and C_4_F_8_ (GOST TU 2412-128-05807960-96). Thus, the basic steps of the fabrication of Si ordered nanostructures using the nanosphere lithography process are shown in Fig. 1.

To understand the process of plasma-chemical etching of polystyrene, it is necessary to study the physicochemical regularities of the processes occurring in plasma. One of the most promising methods for this purpose is in situ control using optical emission spectroscopy (OES). In addition, the OES method was used to determine the end point of the process of removing the nanosphere residues in oxygen plasma by the disappearance of spectral lines specific for polystyrene from the spectra. Optical emission spectra were recorded using an OceanOptics HR 4000 spectrometer in the wavelength range of 200–1120 nm with a resolution of ~ 0.02 nm. The spectrometer was connected to the reaction chamber through a fiber-optic cable, which was integrated with the viewing window on the flange of the reaction chamber of the PCE system. Post-processing of the data was performed using SpectraGryph 1.2.14 software.

To determine the concentration of atomic oxygen in plasma, the method of optical actinometry was applied, for which the emission line of O (844.6 nm) and argon (750.4 nm) as the gas-actinometer were used. The equation for the concentration of atomic oxygen can be written as follows:

$$\left[O\right]={K}_{O}\frac{{I}_{O}}{{I}_{Ar}}[Ar],$$

where I_O_ and I_Ar_ are the experimentally recorded emission intensities of oxygen (844.6 nm) and argon (750.4 nm) lines respectively, [Ar] is the argon density inside reactor without plasma discharge, K_O_ is the oxygen excitation efficiency coefficient (0.07)^32^.

**Figure 2:**
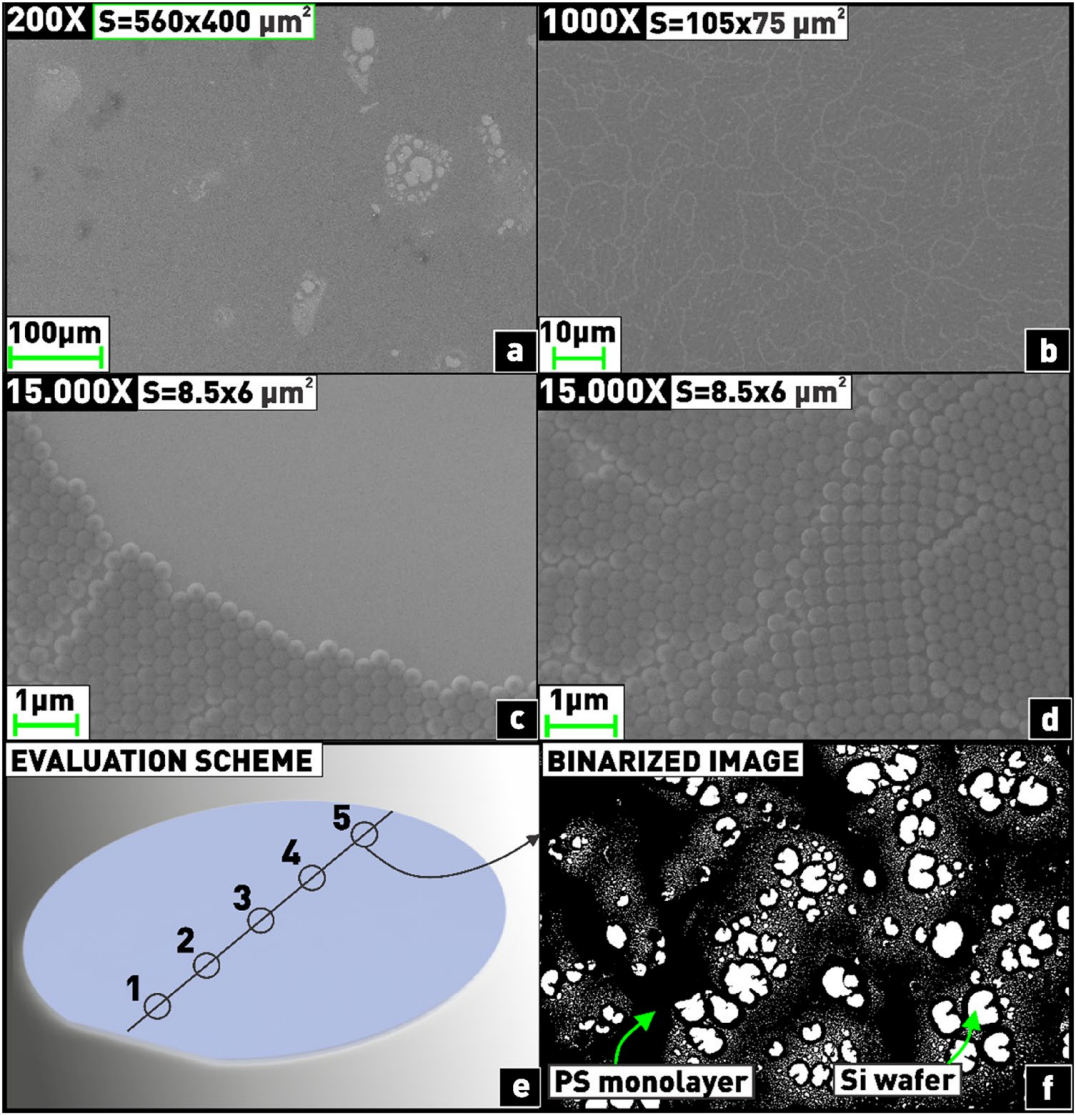
Methodology for estimating nanosphere coverage area: **(a–d)** demonstration of differences in the results obtained at different magnifications; **(e)** five-point measurement scheme for coverage area; **(f)** binarization of the image for coverage area estimation.

After conducting the processes of nanosphere lithography and plasma-chemical etching, the resulting coatings of polystyrene nanospheres and Si nanostructures were analyzed using microphotographs made with a Supra 55VP (CarlZeiss) scanning electron microscope with an accuracy of ± 2.5%. As shown in the literature review, in most of the works, the coverage area of the substrate with mono- and bilayer nanospheres was estimated by microphotographs covering the substrate area not exceeding 100 × 100 μm^2^^19,20,22,23,26^. The microphotographs in Fig. 2a–d show that high magnification does not give an objective result regarding the overall quality of the nanosphere coating. For example, a microphotograph at 1000 × magnification, covering an area of 105 × 75 μm^2^ (Fig. 2b), shows 100% filling of the substrate with a monolayer of nanospheres. However, if the area is enlarged to 560 × 400 µm^2^ (Fig. 2a), the presence of both voids (lightest regions) and double-layered areas (darkest regions) can be observed, shown separately in Fig. 2c,d. Therefore, in order to provide visual reliable information, in this work the analysis of the resulting coatings was conducted using microphotographs taken at a magnification of 200x (560 × 400 µm^2^). The coverage area of the substrate with nanospheres was estimated from a histogram of the binarized image, as shown in Fig. 2f (the nanospheres are painted black, the voids—white). Each experiment was carried out 3 times, and 5 points were measured on each substrate (Fig. 2e), after which the obtained values were averaged. Error bars have been set as follows: ± 2.5% for the dependencies of coverage areas on various parameters and ± 5% for the dependencies of nanosphere etching rates on various parameters.

In addition to the desired close-packed monolayers with hexagonal structure, the obtained nanosphere arrays may contain voids (uncovered regions), locally arranged monolayers with random ordering, and borders between domains. Domains will be called regions of the array with high ordering and hexagonal symmetry. In this regard, to check the hexagonal symmetry of the resulting self-assembled monolayer, a 2D Fourier transform was applied to the SEM image in the NT-MDT NOVA software. In order to estimate the size of domains in the array obtained using the developed technique, the HEXI software^33^ was used. It recognizes regions with hexagonal ordering in the loaded SEM image and marks the borders between them, as well as defects in the domains in the form of voids.

## Results and discussion

Theoretical studies^34,35^ have demonstrated that interparticle capillary forces are the driving force for the ordering of nanospheres on the substrate. Such forces occur as a result of increasing curvature of the liquid surface between the particles as the solvent evaporates. As a result of the action of capillary forces, the nanospheres are assembled into a hexagonal close-packed structure due to the fact that the system tends to the configuration with the lowest energy, and hence, to the maximum contact with neighboring particles. Thus, the evaporation rate of the solvent, consisting in our case of isopropyl alcohol and propylene glycol, is one of the main factors, which affects capillary forces and, as a result, the process of self-assembly of nanospheres^36^. In turn, the evaporation rate of the solvent is influenced by parameters such as rotation rate (N) and time (T), as well as the viscosity of the solution, which depends on the relative concentrations of each component in solution (V_1_—isopropyl volume, V_2_—propylene glycol volume, V_3_—volume of aqueous solution of nanospheres). In this regard, the nature of the influence of the above technological parameters on the area of the substrate covered by mono- and bilayer of nanospheres was studied in order to determine the optimal technological parameters for creating a monolayer close-packed nanosphere coating.

The content of each component in the solution applied to the substrate was considered as a percentage of the volume of one component to the total volumes of the other two components. This was done for the convenience of information perception, since in a series of experiments to determine the optimal contents of nanospheres, alcohol and propylene glycol in the solution, the total volume of the solution applied to the substrate varied according to the varying volume of the component under consideration, while the volumes of the other two components were unchanged.

The initial volume ratio of all solution components was determined during a preliminary series of experiments aimed at optimizing the parameters of the nanosphere lithography process based on the Taguchi matrix method^37^. The best result in terms of substrate coverage area was shown by the V_1_/V_2_/V_3_ = 80/56/50 μl solution, which was chosen as starting solution for further basic experiments. The influence of the time and temperature of ultrasonic mixing of the solution on the quality of the coating was also investigated, during which the best quality coatings were obtained at 15 min and 50 ℃, respectively. The ultrasonic treatment parameters were constant in all subsequent series of experiments.

**Figure 3:**
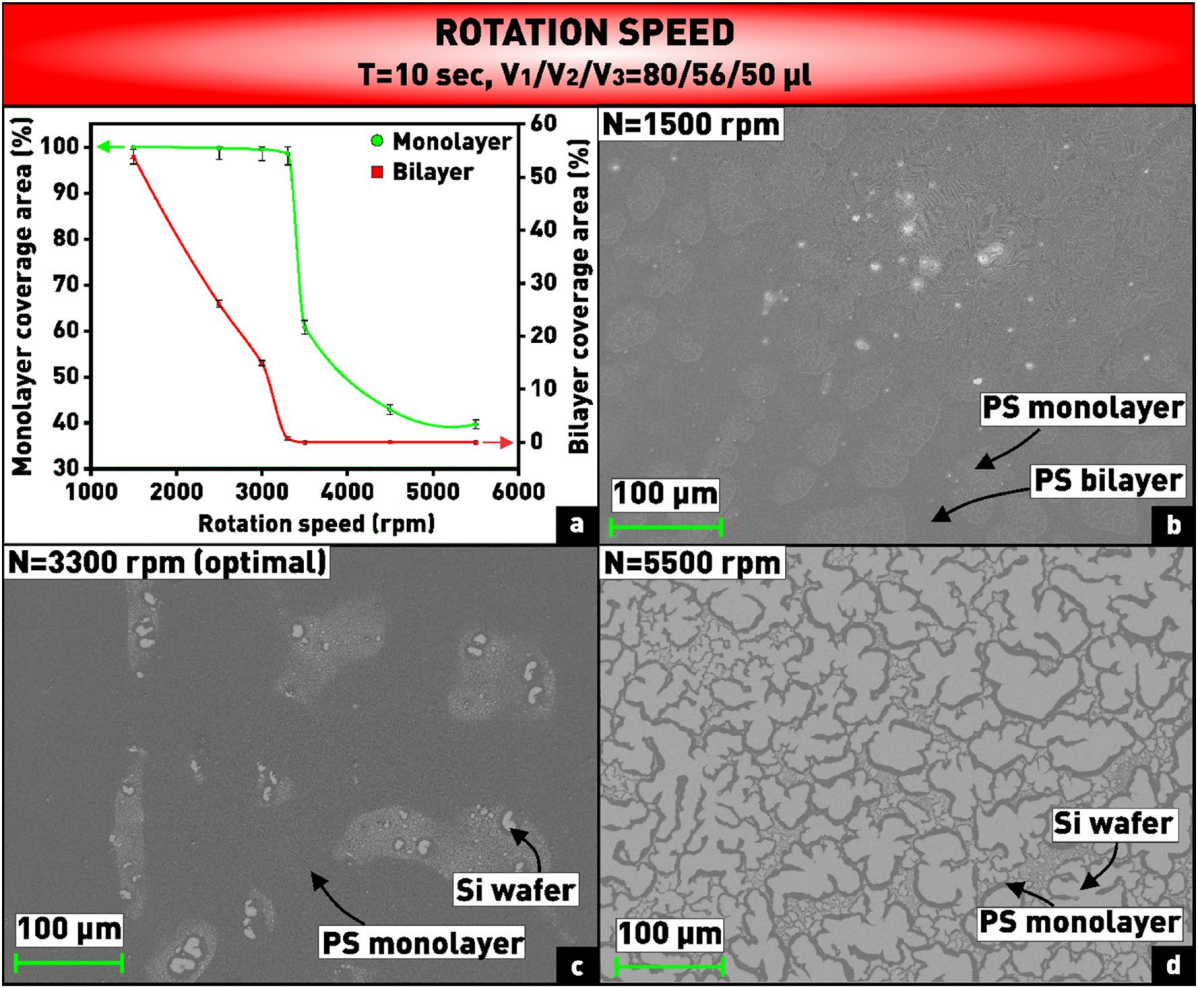
**(a)** Plot of dependence of substrate coverage area by monolayer and bilayer of PS nanospheres on spin speed; **(b–d)** microphotographs of nanosphere coatings obtained at varying spin speed.

The first series of experiments was aimed at determining the nature of the influence of the spin speed on the quality of the coating, while other process parameters remained unchanged. It is known that the spin speed affect both the solvent evaporation rate and the centrifugal force, which moves the suspension to the edge of the substrate^20^. As can be seen from the plot (Fig. 3a), at a rotation speed below 3300 rpm, a significant number of bilayer clusters are formed, shown in the microphotograph in Fig. 3b. This double-layered areas are not acceptable for the fabrication of nanosphere coatings used as masks for subsequent etching. This is probably due to the fact that at low spin speeds the balance of forces acting on the nanospheres is disturbed. Due to the low centrifugal force, the flow of nanospheres toward the edge of the substrate is not sufficient to compensate the capillary flow of the suspension into the central region. On the contrary, the high spin speed (> 3500 rpm), which facilitates the evaporation process and increases the centrifugal force, creates a large number of voids in the array (Fig. 3d), because most of the suspension is thrown away from the substrate surface. In addition, the solvent evaporates faster than the nanospheres have time to self-assemble into a hexagonal array^22^. The optimal spin speed of 3300 rpm was found experimentally, at which the coverage area was 98.6%, and there were almost no bilayers (0.7%) (Fig. 3c). With further increase of the spin speed, the bilayers completely disappear, but the total coverage area of the substrate decreases sharply.

In the next series of experiments, we determined the optimal amount of aqueous solution of nanospheres, which was taken from a purchased solution and centrifuged. Then the water was removed from it, and finally the dry spheres were mixed with solvent in the form of isopropyl alcohol and propylene glycol.

**Figure 4:**
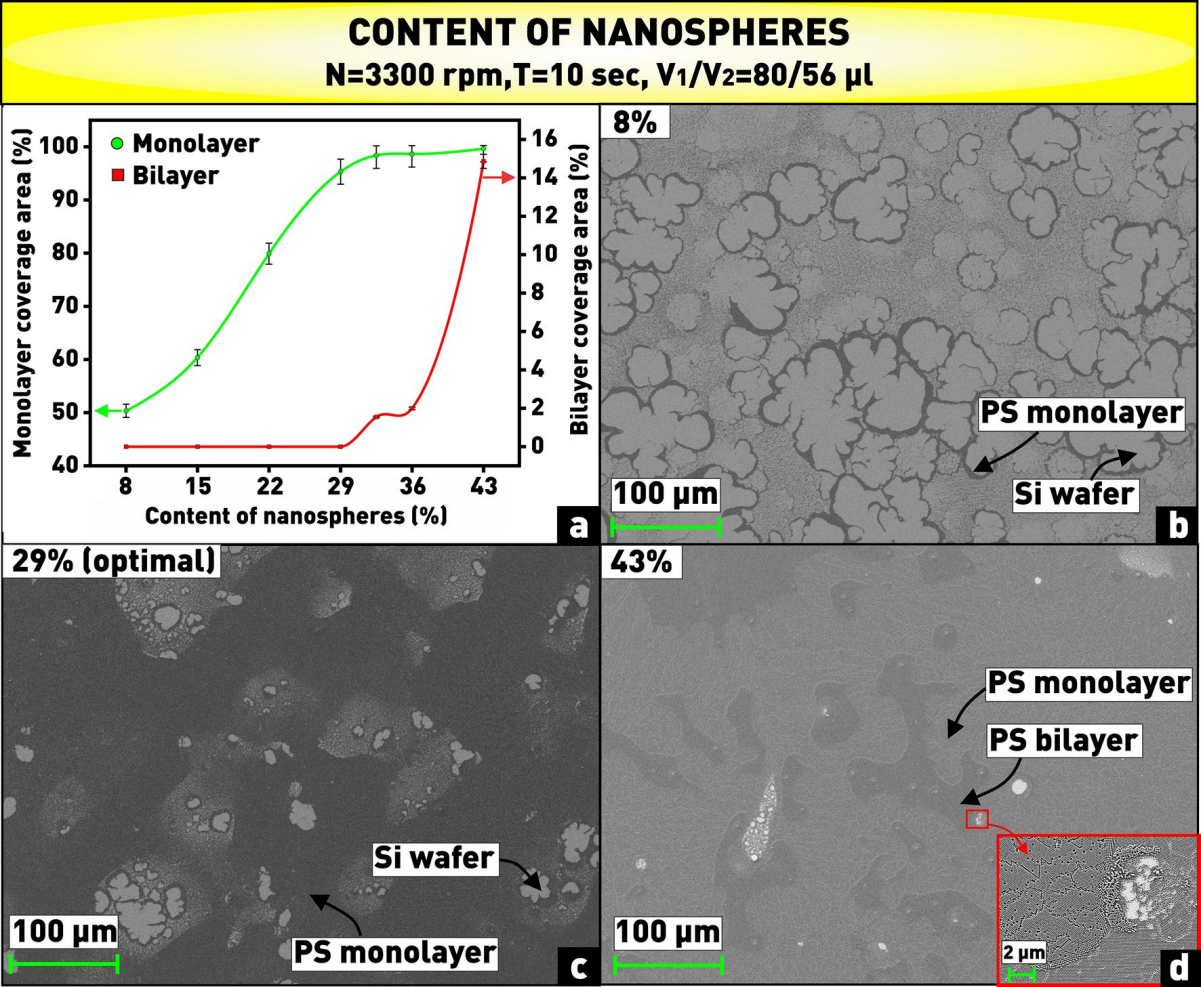
**(a)** Plot of dependence of substrate coverage area by monolayer and bilayer of PS nanospheres on content of nanospheres in solution; **(b–d)** microphotographs of nanosphere coatings obtained at varying content of nanospheres in solution.

As can be seen from the plot (Fig. 4a), a good coverage area close to 100% is achieved at nanosphere content (in this case the percentage ratio of the volume of the aqueous nanosphere solution discussed above to the total volume of isopropyl and propylene glycol) starting from 29%. Smaller content values produce a large number of empty spaces on the substrate, as can be seen in Fig. 4b. However, the number of bilayers begins to increase sharply at nanosphere content of 37% and higher. This indicates that at the selected technological parameters of the spin speed and time, a larger amount of polystyrene remains on the substrate than is necessary for the close-packed monolayer coating (Fig. 4d). Therefore, a nanosphere content of 29% was selected for further experiments, corresponding to the volume of the initially sampled aqueous nanosphere solution of 40 µl. At this chosen value, the coverage area was equal to 95.3%, and there were no bilayers (Fig. 4c).

**Figure 5:**
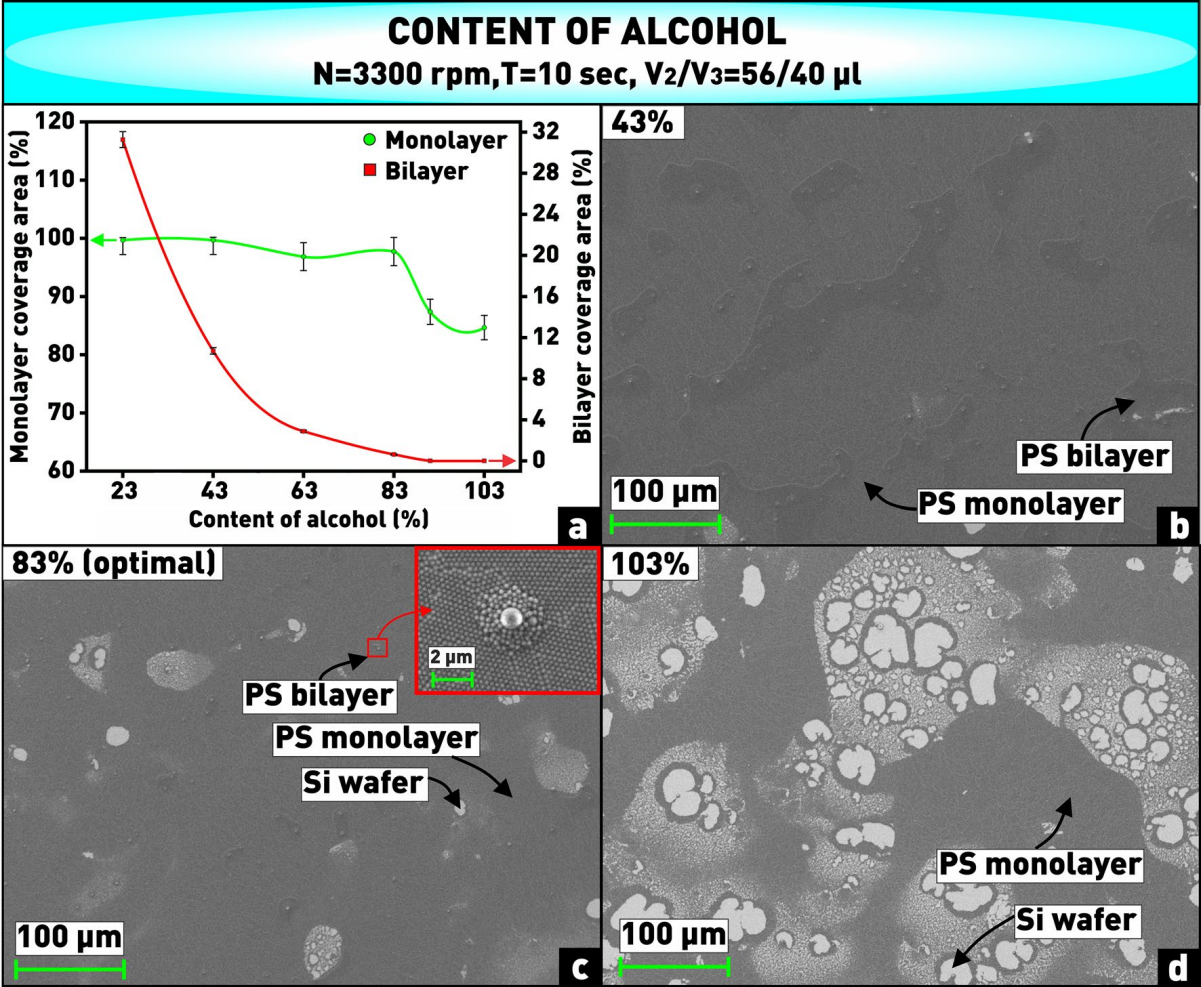
**(a)** Plot of dependence of substrate coverage area by monolayer and bilayer of PS nanospheres on content of isopropyl in solution; **(b–d)** microphotographs of nanosphere coatings obtained at varying content of isopropyl in solution.

The viscosity of the solution also has a significant effect on solvent evaporation and self-assembly of the nanospheres, and hence on the coverage area and the number of bilayers. Solution viscosity can be controlled by changing the relative concentrations of isopropyl alcohol and propylene glycol in the solution. Nanospheres without solvent are not capable to self-assemble into a dense hexagonal package, because there are electrostatic repulsive forces between them, which are overcome by surface tension forces arising from solvent carriers in the colloidal solution^38^. In addition, propylene glycol, which has two O–H groups and a small hydrophobic chain, wets the polystyrene surface more strongly than water and isopropyl alcohol, so it reduces the hydrophobic interactions of nanospheres in the solvent, which prevent the processes of reorientation and compaction of particles^39^. As can be seen from the plot (Fig. 5a), increasing the content of isopropyl alcohol (in this case the percentage ratio of the alcohol volume to the total volume of nanospheres and propylene glycol) from 23 to 103% leads to decreasing the total coverage area of the substrate, while the number of bilayer regions also decreases and is close to 0 at the content of isopropyl more than 83%. This is probably due to the fact that the small volume of isopropyl, which has a viscosity slightly higher than a viscosity of water, evaporates very quickly. Consequently, only propylene glycol with much higher viscosity remains in the solution, and it becomes more difficult for the nanospheres to move across the substrate surface with such excessive solution viscosity. As a result, more than one monolayer remains on the substrate (Fig. 5b). Increasing the content of isopropyl in the solution leads to a more rarefied and non-uniform coating (Fig. 5d). Due to decreasing the relative concentration of propylene glycol, the compaction forces are not capable to overcome the barrier of hydrophobic interactions to order the particles, as a result of which the nanospheres are randomly fixed on the substrate. Thus, the optimal value of the isopropyl content in the solution was found to be 83%, corresponding to the alcohol volume of 80 µl. At this chosen value, the substrate is filled with a close-packed array by 97.6%, and there are approximately 0.6% bilayer areas (Fig. 5c).

It is worth noting that a purchased solution of nanospheres of a given size, although in small quantities, initially contains large polystyrene spheres. Therefore, in some cases, such as shown in the inset in Fig. 5c, this single large sphere can act as a nucleation center for multilayered areas of small spheres around it. Such areas have also been included in the total percentage of bilayer coverage of the substrate for a more objective study.

**Figure 6:**
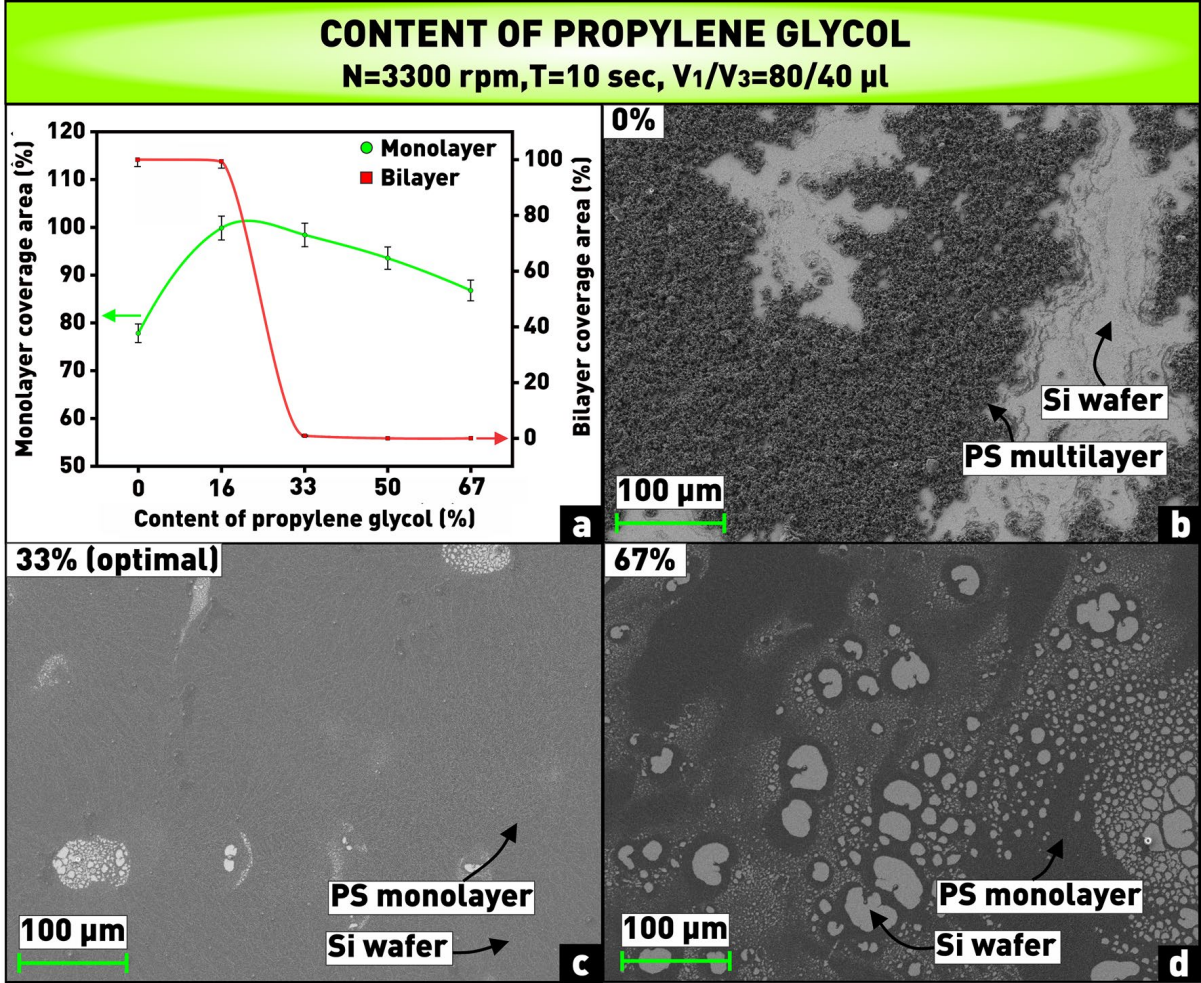
**(a)** Plot of dependence of substrate coverage area by monolayer and bilayer of PS nanospheres on content of propylene glycol in solution; **(b–d)** microphotographs of nanosphere coatings obtained at varying content of propylene glycol in solution.

It follows from the plot in Fig. 6a that the total lack of propylene glycol in the solution leads to a low total coverage area of the substrate (≈ 78%), with the formation of a completely non-monolayer coating. This is probably related to the impossibility of self-assembly of nanospheres due to their poor wettability by isopropyl and the strong effect of hydrophobic interactions between the nanospheres in the solvent without adding propylene glycol to the solution. This assumption is confirmed by the microphotograph (Fig. 6b) demonstrating the formation of multilayer disordered regions of nanospheres in the absence of propylene glycol in the solution. It can also be seen from the plot (Fig. 6a) that with increasing the propylene glycol content (in this case the percentage ratio of the propylene glycol volume to the total volume of nanospheres and alcohol) from 16 to 33%, there is a slight decreasing coverage area of the substrate by a monolayer of nanospheres and almost complete disappearance of bilayer areas. In this regard, the content of 33% was chosen as the optimal value of the propylene glycol content in the solution, corresponding to the propylene glycol volume of 40 µl, at which the coverage area of the substrate with a close-packed monolayer of nanospheres is 98.5%, and there are approximately 0.8% of bilayer areas (Fig. 6c). As the propylene glycol content is further increased, solution viscosity probably becomes too high for the normal self-assembly process, and a significant number of voids appear, as shown in Fig. 6d.

**Figure 7:**
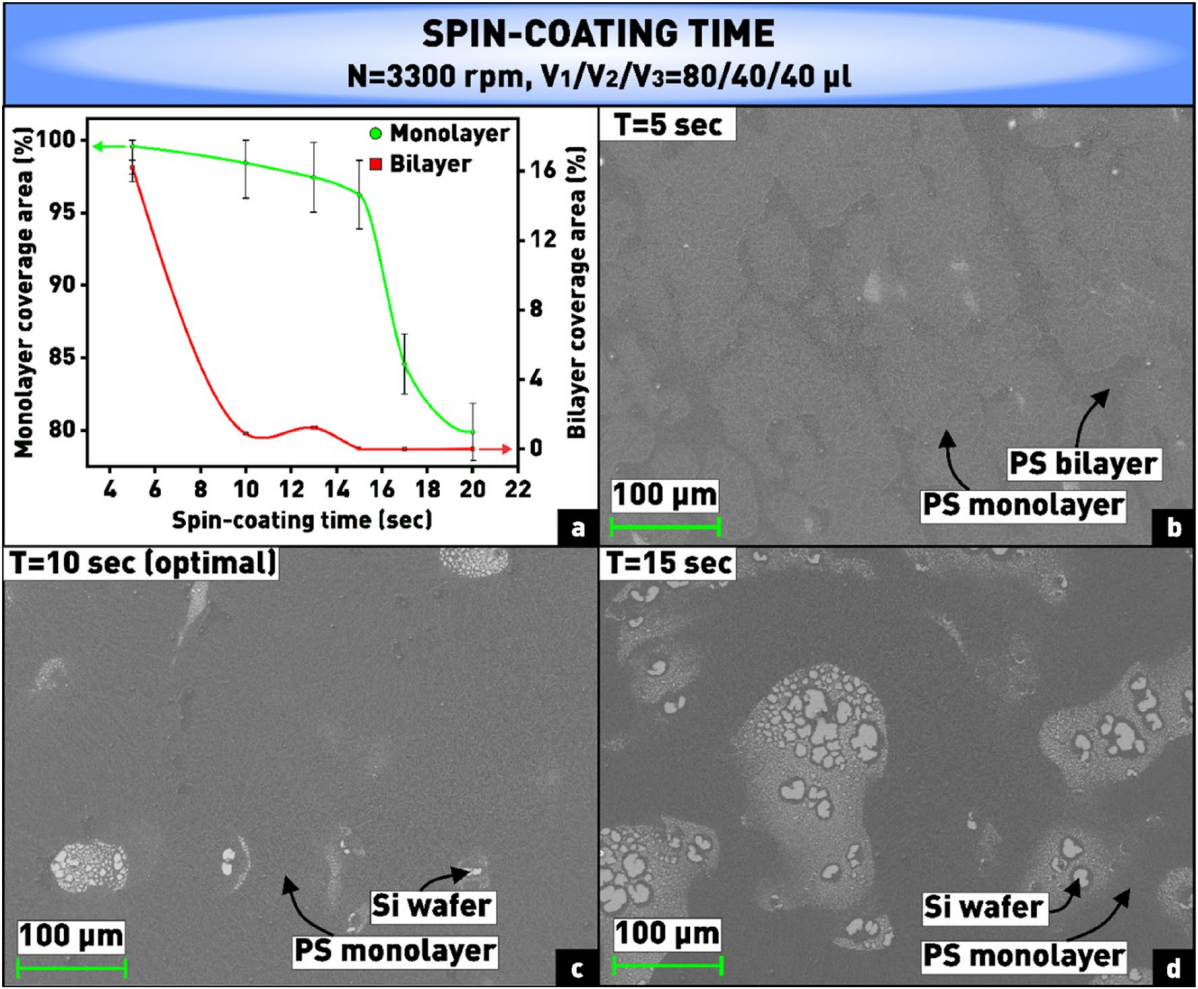
**(a)** Plot of dependence of substrate coverage area by monolayer and bilayer of PS nanospheres on spin-coating time; **(b–d)** microphotographs of nanosphere coatings obtained at varying spin-coating time.

The final series of experiments was aimed at determining the nature of the influence of spin-coating time on the coverage area of the substrate with mono- and bilayer of nanospheres. According to the obtained plot shown in Fig. 7a, when the rotation time is less than 10 s, a significant number of bilayer areas are formed, demonstrated in Fig. 7b. This is due to the fact that the bulk of the suspension still remains on the substrate, and the solvent does not have time to evaporate. Moreover, in addition to the bilayers, a short rotation time during the spin-coating process formed a thickening on the periphery of the substrate, which flowed over a large area of the substrate and reduced its working area. On the contrary, at spin-coating times longer than 10 s, as follows from the plot (Fig. 7a) and the microphotograph (Fig. 7d), the formation of voids was observed, because by this time, probably, too many nanospheres leave the substrate. Using the obtained dependences, the optimal spin-coating time was chosen to be 10 s (Fig. 7c).

The optimal technological parameters of the nanosphere lithography process for obtaining the close-packed hexagonal array of polystyrene nanospheres with a diameter of 300 nm were determined on the basis of analyzing the obtained experimental data (Table 1). To check the reproducibility of the results, five experiments with the selected optimal parameters were conducted, and the coverage area with mono- and bilayer was estimated for each (Table 2). As a result, the average coverage area of the substrate with monolayer of the nanospheres (diameter 300 nm) was 97.8%, with a small number of bilayer regions (0.5%).

**Table 1:** Values of technological parameters of control experiments.

N, rpm	T, s	V_1_, μl	V_2_, μl	V_3_, μl
3300	10	80	40	40

**Table 2:** Coverage areas with mono- and bilayers in control experiments.

No	1	2	3	4	5	Average
Area of monolayer, %	98.3	96.4	99.2	96.3	98.7	97.8
Area of bilayer, %	0.5	0.1	0.9	0	0.9	0.5

To estimate the accuracy characterizing the reproducibility of the results, the relative root-mean-square was calculated according to the formula (1)

1 $$\nu =\sqrt{\frac{\sum_{i}^{n}{({x}_{i}-\overline{x })}^{2}}{{\overline{x} }^{2}\cdot (n-1)}}\cdot 100\%$$

where ${x}_{i}$ is the monolayer coverage area of the substrate in each experiment, $\overline{x }$ is the average value of the monolayer coverage area of the substrate, and *n* is the number of experiments, equal to 5. Thus, the relative root-mean-square error was 1.37%, which can be considered quite acceptable result.

**Figure 8:**
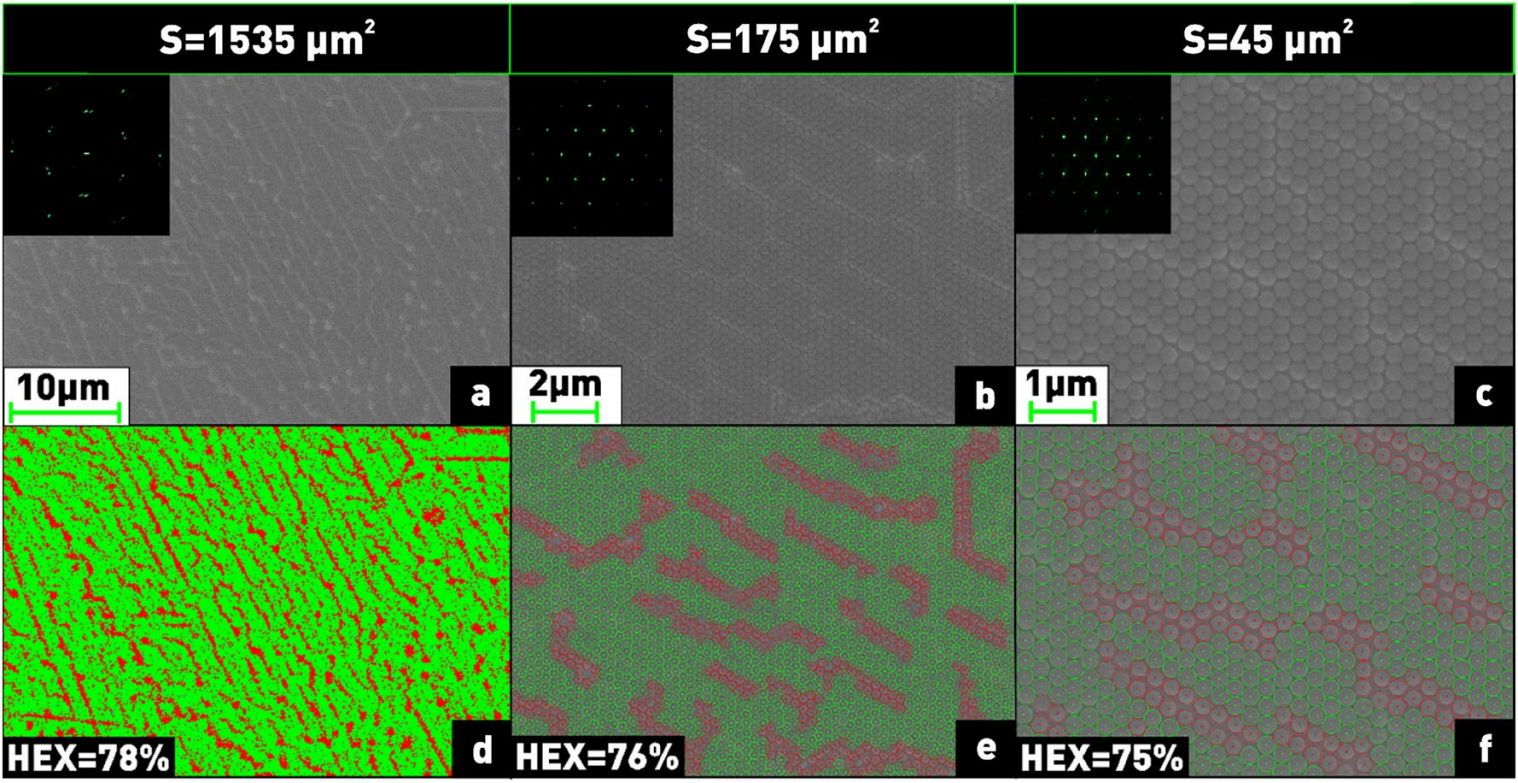
Qualitative and quantitative analysis of the hexagonal lattice on different substrate areas (**a–c**) using Fourier transform and HEXI processing.

It is well known that self-assembled arrays are characterized by individual domains, or as they are called, grains, which can be differently oriented, but within which spheres are strictly ordered into a hexagonal lattice with one orientation. In order to estimate the degree of ordering of the resulting coatings, a Fourier transform of SEM images covering different areas was performed. The corresponding 2D Fourier images of the arrays are shown in the insets in Fig. 8a–c. The maximum covered area, where a hexagonal ordered domain with one orientation was obtained, was found to be 1535 μm^2^, as confirmed by the peaks in the Fourier image located at the vertices of the regular hexagon (Fig. 8a). However, despite the ordering, dislocations can also be present in one domain and are clearly visible in SEM images at high magnification (Fig. 8b,c). Using the HEXI software, the percentage of defective areas in each image was calculated. Close-packed nanospheres are marked with green circles and nanospheres entering dislocation areas are marked with red circles (Fig. 8d–f). The resulting percentage of dislocations was 22% in an area of 1535 µm^2^ (Fig. 8d), 24% in an area of 175 µm^2^ (Fig. 8e) and 25% in an area of 45 µm^2^ (Fig. 8f).

The next step to obtain an array of silicon nanostructures of a certain geometry is the process of reducing the size of polystyrene nanospheres in inductively coupled oxygen plasma, to form an ordered, not close-packed array with gaps between nanospheres. For controlled reduction of nanosphere size in oxygen plasma, it is necessary to study physicochemical regularities of polystyrene etching process, as well as processes occurring in plasma during etching. In this regard, the nature of the influence of the main process technological parameters (high-frequency (HF) power, etching time, bias voltage on the substrate holder, pressure in the chamber, and oxygen flow rate) on the etching rate and the diameter of nanospheres was determined.

**Figure 9:**
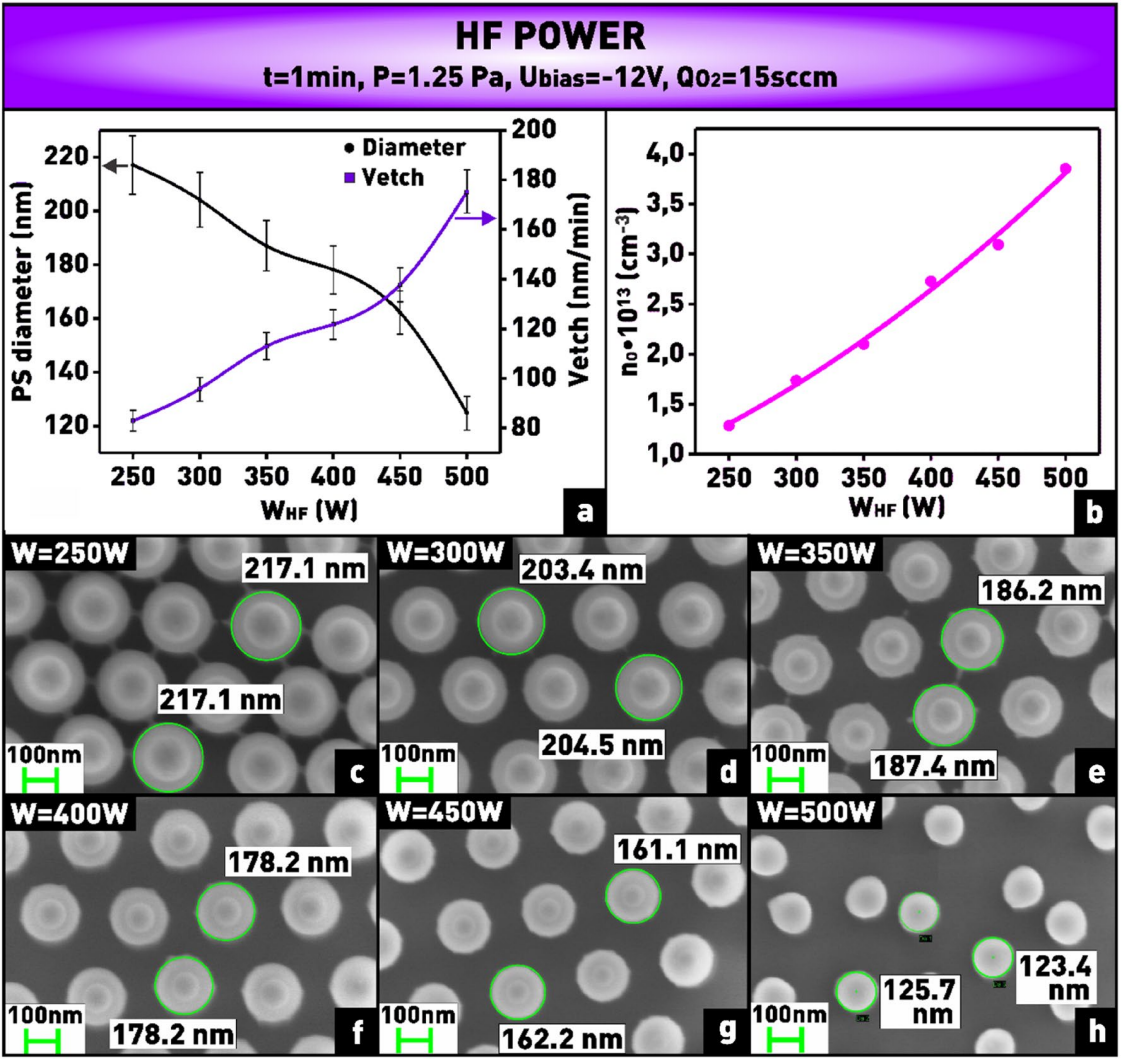
**(a)** Plot of dependence of nanosphere diameter and etching rate on applied HF power; **(b)** plot of dependence of oxygen atom concentration on applied HF power; **(c–h)** microphotographs of nanosphere mask obtained at varying HF power.

The etching of polystyrene in oxygen plasma occurs by breaking the aromatic ring, formation of oxygen-containing functional groups on the surface, and subsequent formation of volatile products such as carbon monoxide and carbon dioxide^40^. Figures 9c–h show how the size of nanospheres etched at different HF power for 1 min changes. From the plot (Fig. 9a) it can be observed that the polystyrene etching rate increases monotonically from 83 to 175 nm/min when the applied power is increased from 250 to 500 W. Such nature of the dependence is related to the fact that with increasing absorbed power, the concentration and average energy of electrons in plasma grows, therefore, the intensity of inelastic collisions of electrons with oxygen molecules increases. As a result, it leads to more efficient formation of such active particles as radicals and ions which react with polystyrene^41^. This is confirmed by the increasing concentration of atomic oxygen in plasma when the applied power is increased (Fig. 9b).

**Figure 10:**
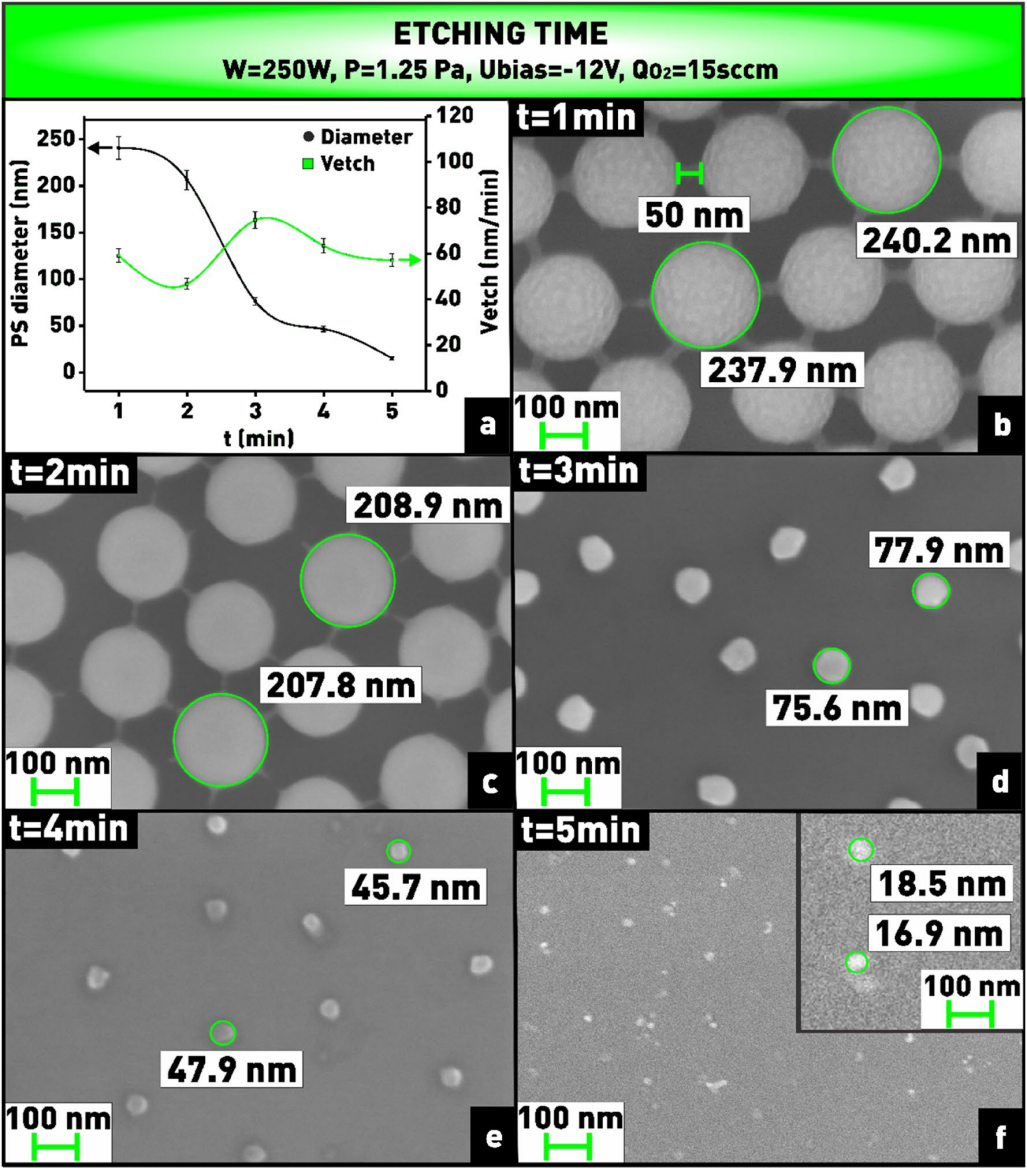
**(a)** Plot of dependence of nanosphere diameter and etching rate on etching time; **(b–f)** microphotographs of nanosphere mask obtained at varying etching time.

Next, the reduction in the size of the nanospheres at different etching times was studied, while the other technological parameters remained constant. As can be seen from the plot (Fig. 10a), the diameter of the nanospheres decreases from 240 to 15 nm when the etching time is increased from 1 to 5 min, while the etching rate changes insignificantly. When the nanospheres are still large enough, during the first 2 min of etching, bridges with a length of about 50 nm are formed between them, thanks to which the nanospheres are well stabilized in their initial positions (Fig. 10b). The formation of such bridges is caused by the tendency of the nanospheres to reduce the surface energy^42^ With further increasing the etching time, these bridges, like the spheres, are etched in oxygen plasma and disappear (Fig. 10c). After that the nanospheres can start moving along the substrate surface, because their size by this time strongly decreases, therefore, the contact area with the substrate also decreases^43^. In addition, as can be seen from Fig. 10d–f, when the etching time reached 3 min, the nanospheres deformed, taking an irregular shape. In this regard, for further experiments the optimal etching time of 2 min was chosen, at which the nanospheres are still well stabilized in their places, are circular in shape and are ≈ 210 nm in diameter (Fig. 10c).

**Figure 11:**
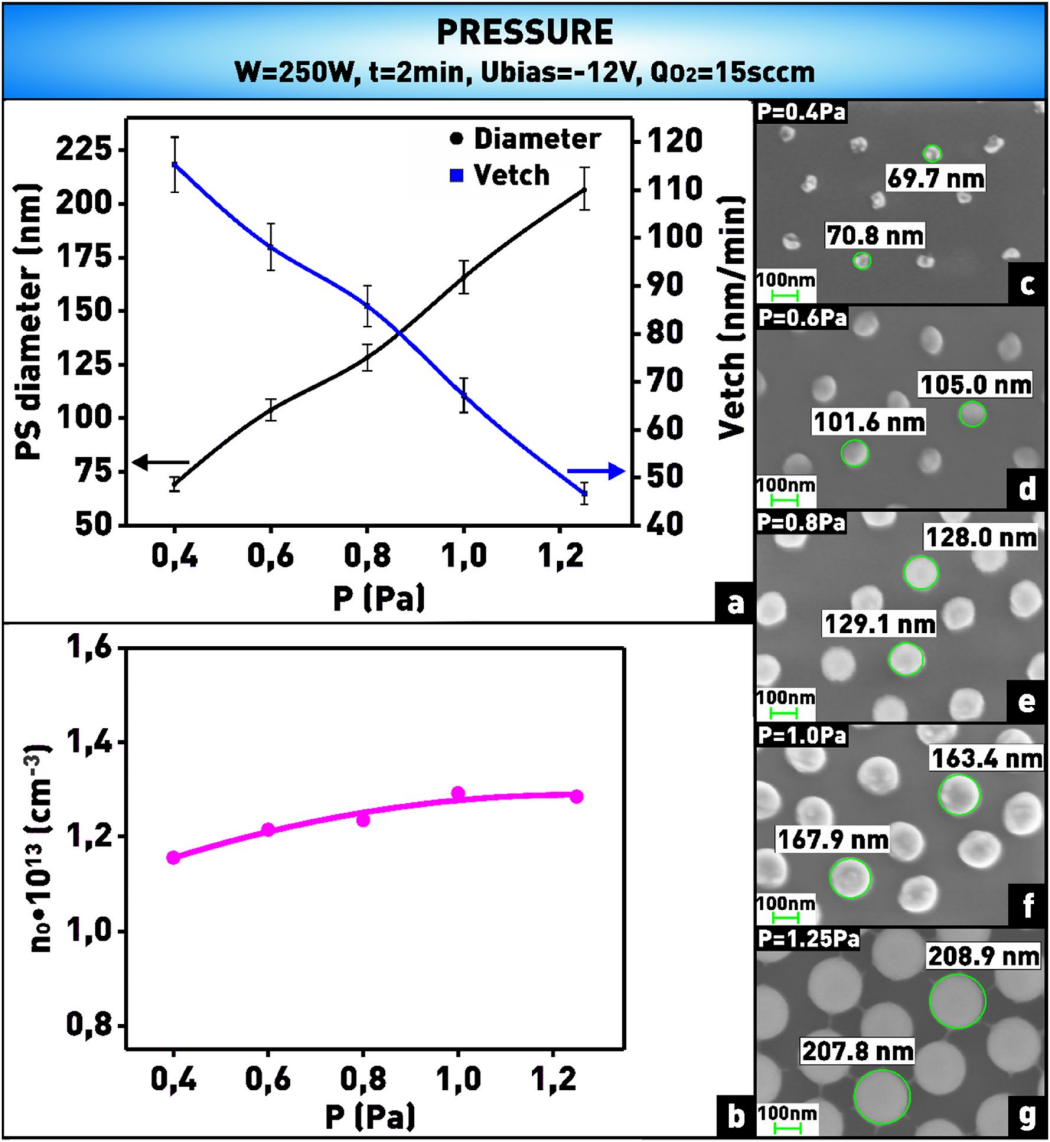
**(a)** Plot of dependence of nanosphere diameter and etching rate on pressure in the chamber; **(b)** plot of dependence of oxygen atom concentration on pressure in the chamber; **(c–g)** microphotographs of nanosphere mask obtained at varying pressure in the chamber.

The pressure in the reaction chamber determines both the energy distribution of electrons and ions and the number of chemical reactions occurring on the surface of the treated material. So, as the gas pressure increases, the energy of ions bombarding the surface decreases due to decreasing the mean free path of the particles. In addition, the average energy of electrons, which determines the rate of generation of active oxygen particles, also decreases^44^. As can be seen from the plot (Fig. 11a) and microphotographs (Fig. 11c–g), the etching rate of polystyrene nanospheres decreased from 115 to 47 nm/min when the pressure in the chamber was increased from 0.4 to 1.25 Pa. However, the dependence of the concentration of oxygen atoms on pressure shown in Fig. 11b demonstrates only a slight increasing atomic oxygen with increasing pressure. Thus, we assume that the contribution of the physical component (i.e., ion bombardment) to the polystyrene etching process prevails in this case over the contribution of the chemical component (i.e., chemical reaction).

**Figure 12:**
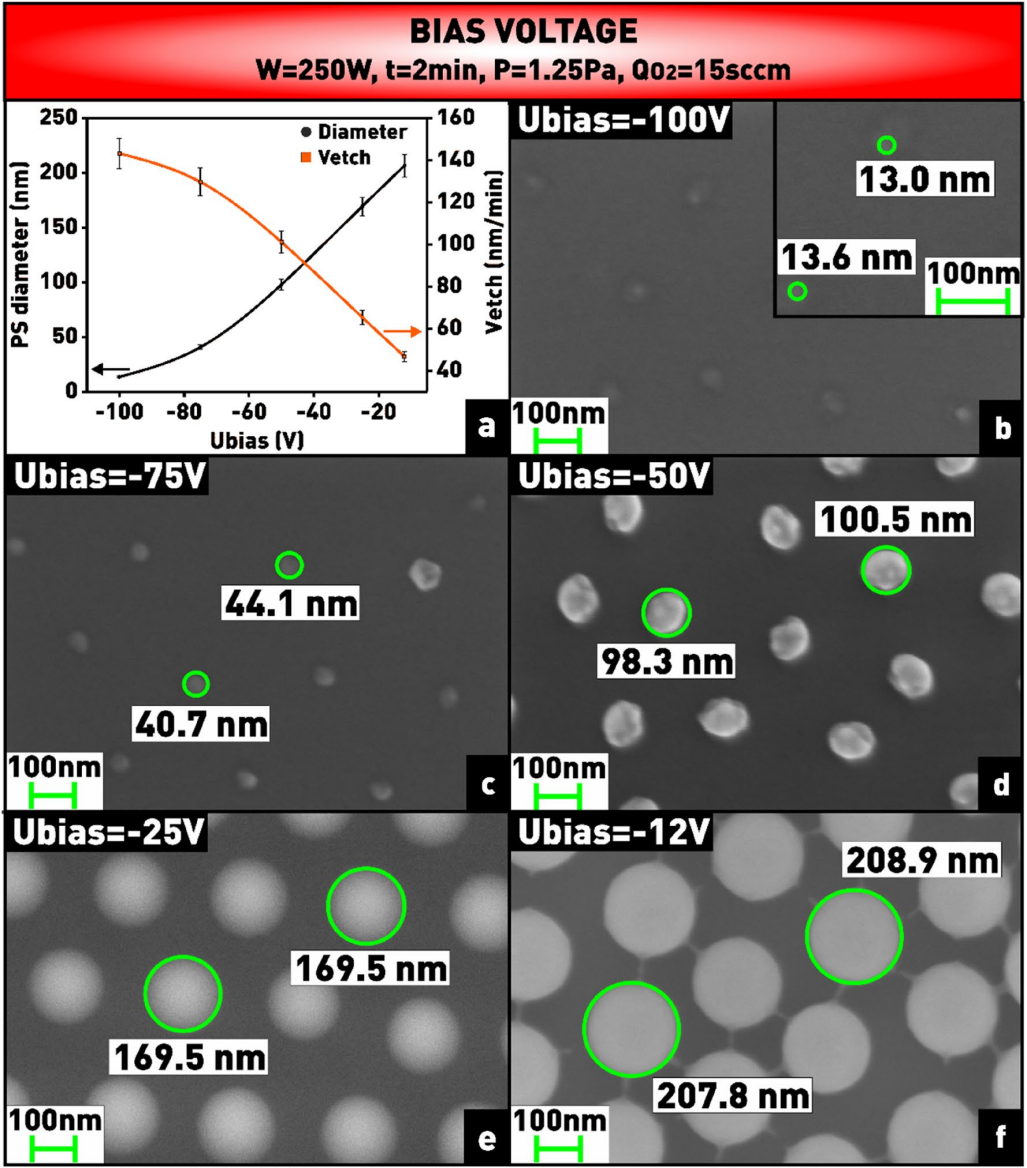
**(a)** Plot of dependence of nanosphere diameter and etching rate on bias voltage on the substrate holder; **(b–f)** microphotographs of nanosphere mask obtained at varying bias voltage.

In addition, decreasing the intensity of ion bombardment of the surface with increasing pressure is confirmed by the fact that at low pressure (< 1 Pa) and higher ion energy the nanospheres become irregularly shaped (Fig. 11c–e). The intensity of ion bombardment is primarily determined by the magnitude of the negative bias potential on the substrate, which also has a great influence on the etching rate and the morphology of the nanospheres^45^. As can be seen from the plot (Fig. 12a), a higher bias voltage leads to a more efficient bombardment of the material, therefore, a higher etching rate. Namely, as the modulus of the bias voltage on the substrate holder increases from 12 to 100 V, the diameter of the plasma-treated nanospheres decreases from 207 to 14 nm (Fig. 12b–f).

**Figure 13:**
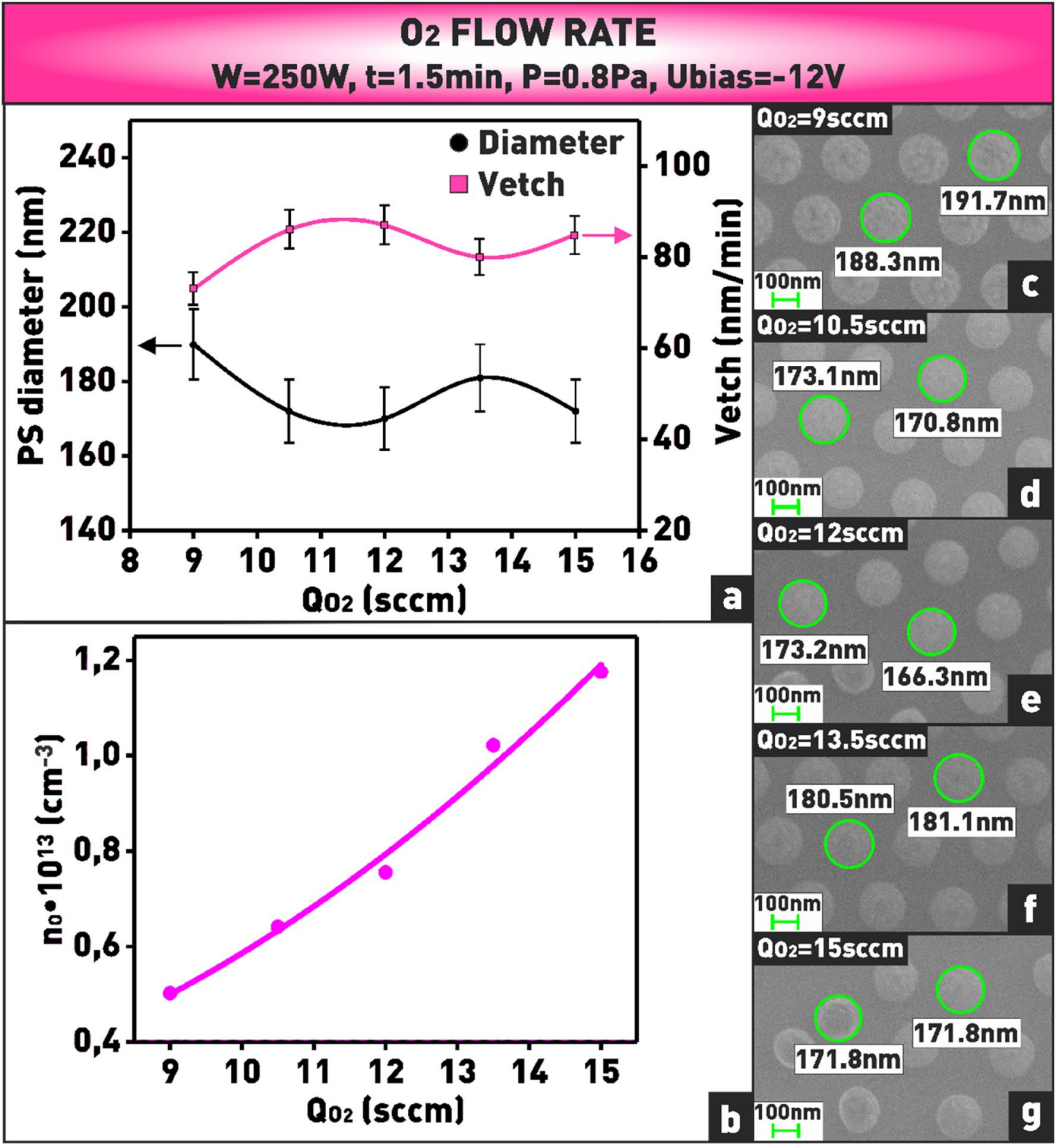
**(a)** Plot of dependence of nanosphere diameter and etching rate on oxygen flow rate; **(b)** plot of dependence of oxygen atom concentration on oxygen flow rate; **(c–g)** microphotographs of nanosphere mask obtained at varying oxygen flow rate.

The final series of experiments was aimed at determining the nature of the influence of oxygen flow rate on the etching rate of polystyrene nanospheres. Despite the fact that the concentration of atomic oxygen increases by approximately two times when the oxygen flow rate is increased from 9 to 15 sccm (Fig. 13b), the polystyrene etching rate does not show significant changes and remains at an average level of 82 nm/min (Fig. 13a,c–g). Probably, in the selected range of technological parameters, the weak variation of the etching rate depending on the oxygen flow rate is related to the fact that there is a certain amount of oxygen sufficient to saturate the chemical etching process^27^. In other words, we assume that in our case the flow rate of 9 sccm is sufficient to saturate the polystyrene surface with functional groups, and a further slight increasing the flow rate to 15 sccm does not significantly affect the probability of interaction of oxygen atoms with carbon atoms in polystyrene.

**Figure 14:**
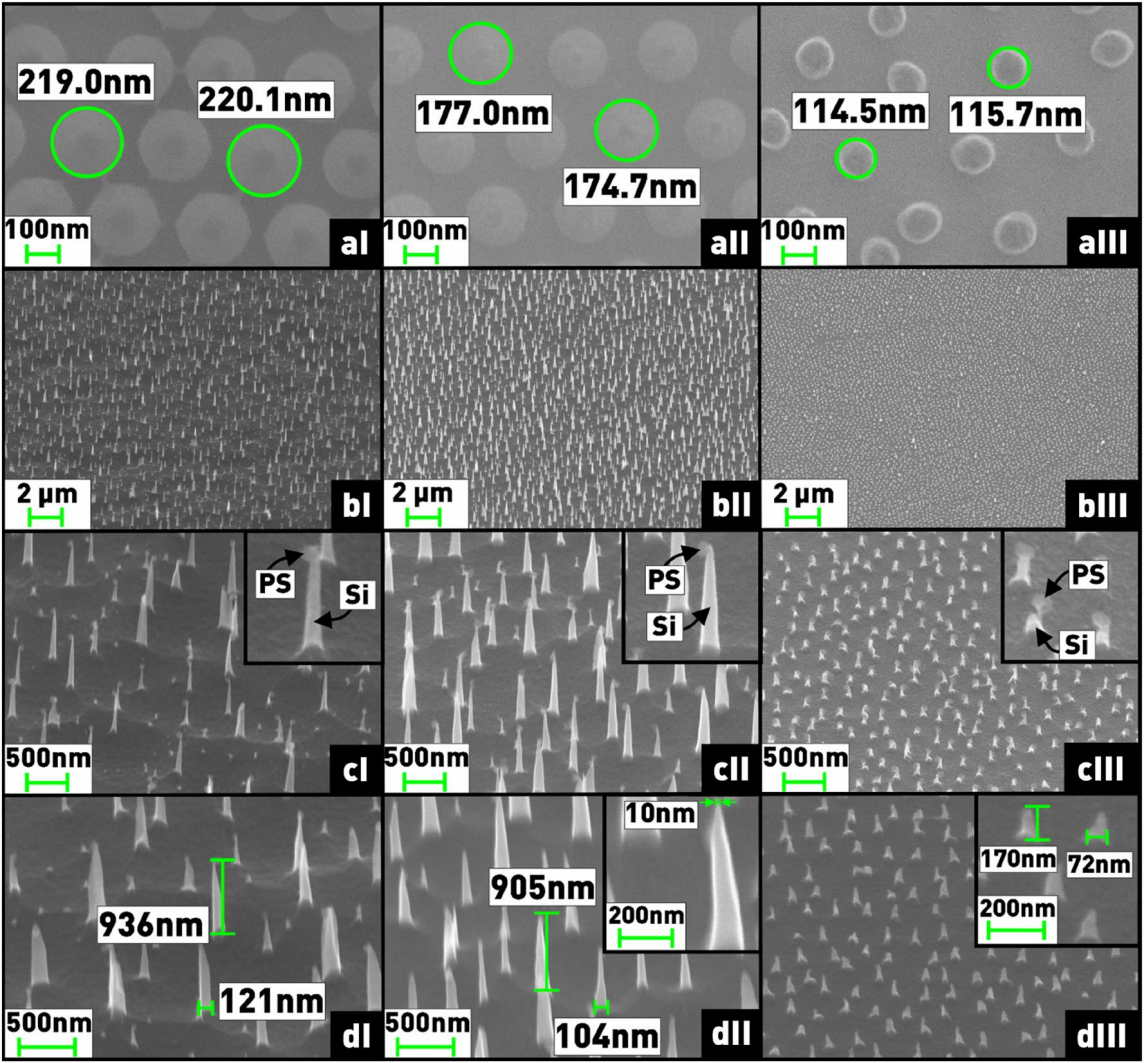
**(a)** Microphotographs of nanosphere masks with different size of nanospheres; **(b,c)** microphotographs of nanoneedle arrays after Si etching in SF_6_/C_4_F_8_ plasma; **(d)** microphotographs of nanoneedle arrays after removal of nanospheres in O_2_ plasma. Microphotographs taken at angle of 45°.

As a result, silicon nanostructures in the form of nanoneedles of various sizes were fabricated using the developed nanosphere lithography technology. For this purpose, at the first stage, ordered close-packed nanosphere masks were formed using spin-coating technique. Then, by PCE in oxygen plasma, the sizes of nanospheres were reduced to different values (220, 175, and 115 nm), as shown in Fig. 14aI–III. Finally, plasma-chemical etching of silicon on the formed mask was conducted in SF_6_/C_4_F_8_ gas mixture, resulting in arrays of nanoneedles with base diameters ranging from 70 to 125 nm, with needle diameters ranging from 10 nm (aspect ratio over 90) to 50 nm, and heights ranging from 170 to 1000 nm depending on the initial size of the nanospheres (Fig. 14bI–III). It should be noted that the dimensions of the nanostructures were estimated as average values from measurements of several needles.

**Figure 15:**
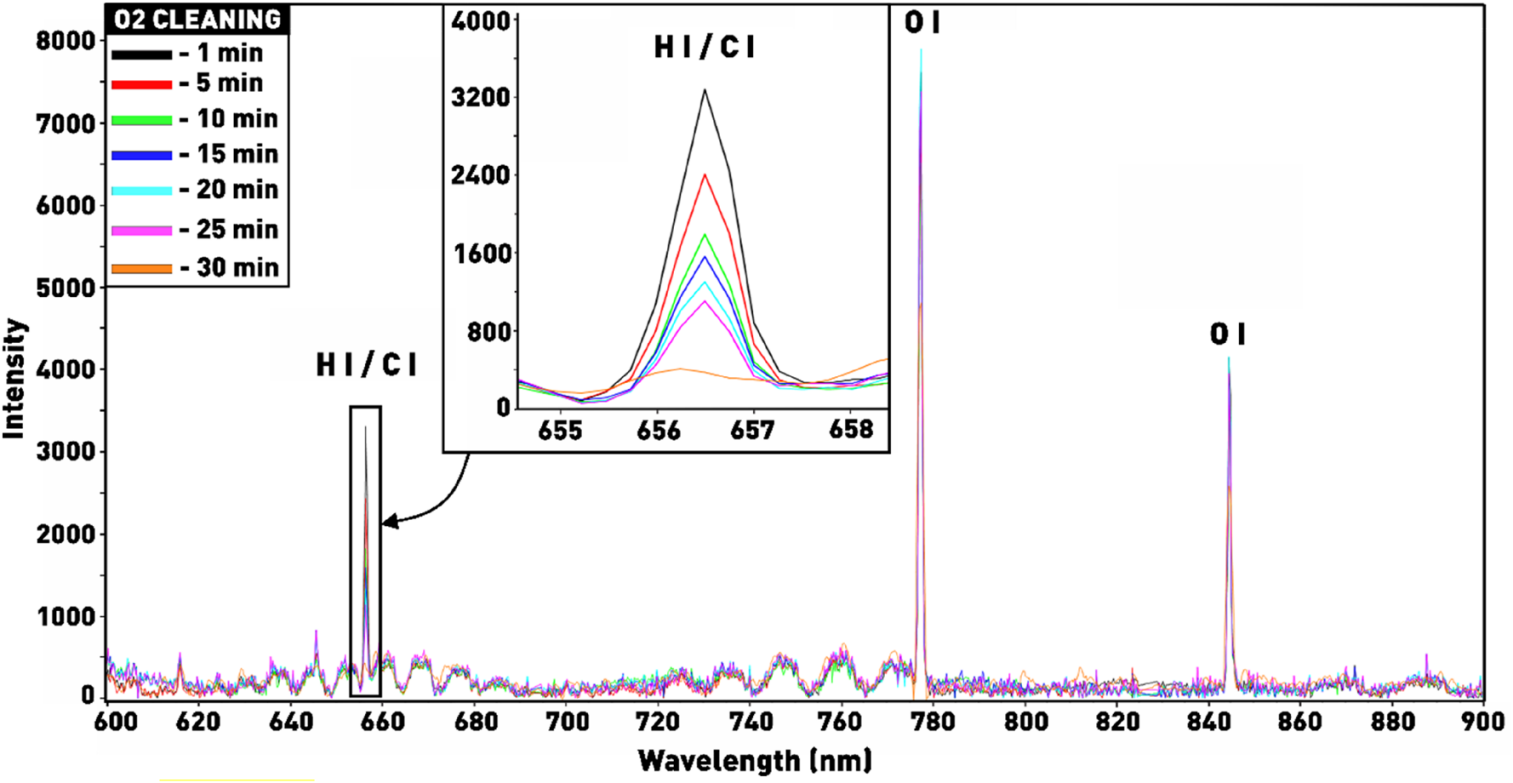
Emission spectra recorded during polystyrene etching in oxygen plasma.

After silicon etching, polystyrene residues still remained on the tops of the nanoneedles (Fig. 14cI–III), which were removed by treatment in oxygen plasma. The optimal time of plasma etching for complete removal of polystyrene nanospheres was controlled in real time using the method of optical emission spectroscopy by changes in the polystyrene-specific spectral lines. Figure 15 shows the plasma emission spectrum during the etching process. We can identify the oxygen lines (844 and 777 nm) and the line belonging to carbon or hydrogen, which have very close values of the emission wavelength (656.28 and 656.87 nm, respectively) and cannot be identified exactly. However, both carbon and hydrogen are formed as a result of polymer chain splitting and atom detachment by active oxygen particles in plasma, therefore, this line can be used to determine the end point of the polystyrene etching process. The time variation of the intensity of the H/C emission line can be clearly seen in the enlarged view in Fig. 15. It can be observed that as the oxygen plasma cleaning time increases, the intensity of the H/C line gradually decreases and approaches 0 when plasma etching reaches 30 min. This indicates the disappearance of reaction byproducts by this time, therefore, the removal of polystyrene nanospheres from the surface of silicon nanostructures (Fig. 14d).

Thus, after the formation of the nanosphere mask, the process of fabricating silicon nanoneedles, which includes reduction of nanosphere size in oxygen plasma, silicon etching and removal of polystyrene residues from the surface of nanostructures, can be conducted in a single technological cycle without additional stages of sample unloading to the atmosphere. In addition, it is possible to use the OES method as in situ control of the end point of polystyrene etching to determine the time of complete removal of polystyrene nanospheres from the sample surface.

## Conclusion

In the course of this study, the technology of spin-coating was developed for creating a monolayer close-packed coating on the substrate using nanospheres with a diameter of 300 nm. For this purpose, the influence of the main technological parameters of the spin-coating process on the solvent evaporation process in the colloidal suspension and, consequently, the self-assembly process of nanospheres on the substrate, was experimentally studied. It was found that at spin speeds less than 3300 rpm, spin-coating time less than 10 s, and contents of isopropyl and propylene glycol less than 83 and 33%, respectively, a significant number of double layers were formed. Increasing the listed parameters above these limiting values resulted not only in the disappearance of bilayers, but also in decreasing the total coverage area of the substrate. An opposite dependence was observed for the content of nanospheres in solution, with the increase of which from 8 to 43% the substrate coverage area increased from 50 to 99.5%. However, similarly there was a limiting value of the content (33%), after which bilayers appeared in addition to the close-packed monolayer of nanospheres. Based on the obtained results, the optimal technological parameters of the nanosphere lithography process were selected, and a monolayer nanosphere coating was obtained with the coverage area of 97.8% on the working area of 76 mm Si substrate and process reproducibility of 98.6%. It should be noted that the selected parameters are suitable for spin-coating using 76 mm Si substrate (in a studied range of technological parameters), but can be extended and used to find conditions for other substrate sizes.

In addition, in this work we showed that using etching of polystyrene nanospheres in inductively coupled oxygen plasma it is possible to easily control the size of nanospheres and distances between them, and, consequently, the size of silicon nanostructures etched through the nanosphere mask. For this purpose, the influence of PCE technological parameters (HF power, bias voltage on the substrate holder, pressure in the chamber, oxygen flow rate, and etching time) on the etching rate of polystyrene nanospheres and their diameter was studied. When HF power was increased from 250 to 500 W, the etching rate pf polystyrene increased from 83 to 175 nm/min, which was confirmed by the increase of the concentration of atomic oxygen in plasma, approximately, by 3 times. On the contrary, the etching rate decreased from 115 to 47 nm/min when the pressure was increased from 0.4 to 1.25 Pa at constant concentration of atomic oxygen. Therefore, it was concluded that ion bombardment made the main contribution to the etching process in this case. As a result, using the developed technology, silicon nanoneedles of various sizes with a maximum aspect ratio of more than 90 were created.

## Data Availability

The datasets generated during and/or analyzed during the current study cannot be shared at this time as the data also forms part of an ongoing study, but are available from the corresponding author on reasonable request.
